# Willingness to Self-Isolate When Facing a Pandemic Risk: Model, Empirical Test, and Policy Recommendations

**DOI:** 10.3390/ijerph17010197

**Published:** 2019-12-27

**Authors:** Xiaojun Zhang, Fanfan Wang, Changwen Zhu, Zhiqiang Wang

**Affiliations:** 1 School of Economics and Management, Fuzhou University, Fuzhou 350108, China; 2 Institute for Risk and Disaster Reduction, University College London, London WC1E 6BT, UK; 3 School of Public Administration, South China University of Technology, Guangzhou 510641, China

**Keywords:** pandemic risk, self-isolation, planned behavior

## Abstract

Infected people are isolated to minimize the spread of pandemic diseases. Therefore, the factors related to self-isolation (SI) should not be neglected, and it is important to investigate the factors leading the infected (or possibly infected) people to choose to self-isolate. In this paper, we tried to show that the theory of planned behavior provides a useful conceptual framework for SI when facing a pandemic risk, and a regression method with Chinese provincial (Guangdong Province) data was applied to investigate how attitude (ATT), subjective norms (SN), and perceived behavioral control (PBC) influence SI when facing a pandemic emergency. The results and the robustness tests confirm that ATT, SN, and PBC have a significant positive influence on SI when facing a pandemic emergency. ATT plays the most important role, followed by SN and then PBC. Based on the factors of SI, we found, through theoretical and empirical analyses, at least three important aspects that local governments need to consider to encourage citizens to self-isolate when facing a pandemic.

## 1. Introduction

Alongside wars and natural disasters, plagues and epidemic (pandemic) diseases have had the highest death tolls in human history [1]. The WHO reported that the next influenza pandemic “is a matter of when, not if” [2], and the world must prepare for the next inevitable flu pandemic. What can we do when the pandemic comes? Outside pharmaceutical interventions, non-pharmaceutical interventions (NPIs) play an important role in delaying the first wave, reducing its peak and the spreading of new influenza cases across time [3,4] because a pandemic vaccine will not be in place when a pandemic starts. NPIs include more actions than just self-isolation (SI), such as quarantining infected populations; the closing of borders, schools, and work places; hand-washing; the cleaning of surfaces; and so on [5]. The isolation of infected people has been applied to minimize the spread of pandemic diseases at least since the Old Testament period as an instrument for controlling and quelling the spread of viruses and contamination agents [6]. Hence, isolation is seen as a critical part of public health interventions, as it protects people by separating those who have been infected by communicable diseases from the general population [7].

Many scholars believe that isolation has a great impact on preventing or delaying the spread of pandemics [8,9]. SI means symptomatic individuals confine themselves to their homes [10]. Generally, isolation can take two forms: Mandatory and voluntary [11,12]. Voluntary SI means that infected (or possibly infected) individuals choose to confine themselves to their homes; this intervention is generally considered capable of limiting the transmission of pandemic influenza [13,14] and is recommended by the European Centre for Disease Prevention and Control [15]. The early initiation of voluntary SI can overcome the negative effects of a delay in antiviral drug distribution when enough symptomatic individuals comply with home confinement at symptom onset [14]. Overall, SI is of great importance for hampering the spread of pandemics and has been widely studied based on different methods. The effectiveness of voluntary SI largely depends on public adherence to this intervention measure [14]. Unfortunately, voluntary SI strategies may inconvenience individuals, lead to economic losses, or even contribute to moral conflicts; thus, voluntary SI remains a controversial strategy [15,16].

To date, many scholars have demonstrated the relationship between SI and the prevention of pandemics [8,10,12,16]. However, the factors involved in SI should not be neglected, and it is important to investigate which factors will encourage infected (or possibly infected) people to choose self-isolation. Surveys conducted in the United States (US) and Australia during the 2009 pandemic showed that more than 80% of people were willing to stay home from work or school [17,18], while 53–76% of people were willing to self-isolate [17,19]. According to the self-reported behavioral intention regarding the H1N1 influenza of university students in southwestern US, Mas et al. (2012) claimed that an array of issues may influence students’ decision to self-isolate, including interpersonal, academic, environmental, and social factors [20]; however, their analysis lacks an empirical basis. Risk perception has been widely established as a significant predictor of engagement in preventive health behaviors, including SI [21]; those who report being unfamiliar with the term “pandemic influenza,” male respondents, and employed people who are not able to work from home have been found to be less willing to comply [22]. A survey in two counties in North Carolina showed that 50% of households with children under 18 and 65% of working adults reported the ability to comply with SI at home for 7–10 days if recommended to do so by the authorities [23]. Concomitantly, recent polls have shown that the willingness to comply with an SI period strongly depends on the social condition and literacy of the individual [24].

Therefore, we seek an answer to the fundamental question about what factors affect the willingness to self-isolate. The remainder of the paper is organized as follows. Section 2 develops a conceptual model to explain the behavior. The data description, empirical results, and regional heterogeneity test are presented in Section 4 and Section 5. Finally, Section 6 presents our conclusions.

## 2. Conceptual Model and Hypotheses

**Figure 1:**
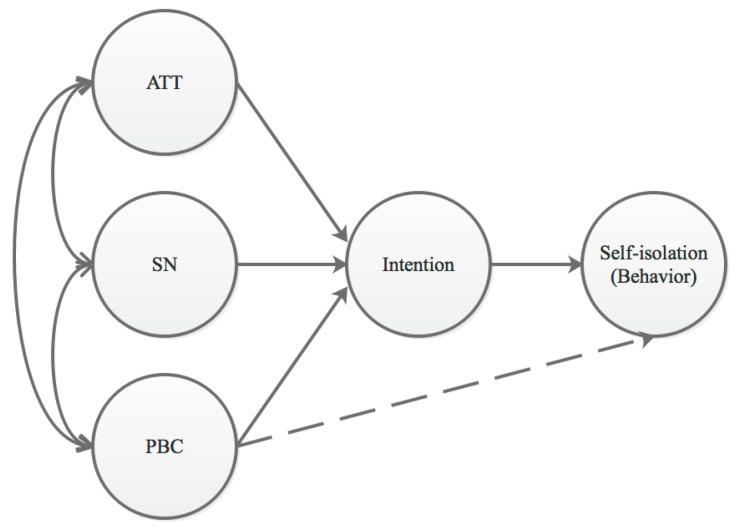
Conceptual Model. ATT, attitudes toward the behavior; SN, subjective norms with respect to the behavior; PBC, perceived control over the behavior.

Several theories explain social behaviors [25]. One is the theory of planned behavior (TPB), which is widely applied to explain many types of behaviors. The TPB is a cognitive theory that provides a useful framework for predicting and identifying health-related behaviors, which are usually found to predict behavioral intentions with a high degree of accuracy. The TPB proposes that the individual is influenced by three factors (see Figure 1): Attitudes toward the behavior (ATT), subjective norms with respect to the behavior (SN), and perceived control over the behavior (PBC) [26,27]. The TPB has been explored in relation to behaviors such as counterproductive work behaviors [28], safe sexual behaviors among drug users [29], consumption of a low-fat diet [30], and healthy eating behaviors [31]. For all of these reasons, it is appropriate to apply the TPB to the study of SI intention when facing pandemic risk. 

In the present study, we sought to build upon the conceptual model and analyze factors correlated with SI behavior based on the TPB. ATT refers to the degree to which a person has a favorable or unfavorable evaluation or appraisal of the behavior in question [26]. The TPB is an extension of the theory of reasoned action [32,33], and the individual’s intention is a central factor of performing a given behavior: When the intention to engage in a behavior is stronger, the behavior is more likely to be adopted [26]. Consequently, the effect of ATT on SI can be positive or negative. SN refers to the perceived social pressure to perform a behavior or not [26,34]. People are sensitive to the conformity pressures associated with real and perceived social norms as related to risk behavior [35]. As many studies have noted, SN and ATT are suggested to exert their effects on behavior through intentions [27]. PBC refers to the perceived ease or difficulty of performing the behavior, and it is assumed to reflect past experience as well as anticipated obstacles [26]. PBC has been found to influence both behavioral intentions and actual behavior [36]. The resources and opportunities available to a person must, to some extent, dictate the likelihood of behavioral achievement [26].

Besides, several other factors may affect the willingness to self-isolate. Gender differences are significant in the attitudes and behaviors related to pandemics [37,38,39]. Here, the factors that affect SI are similar to those identified at the beginning of a pandemic, such as H1N1, HIV, and SARS, suggesting that gender has an effect on SI [40]. A strong intention to comply with government-advised preventive measures in the future is associated with higher age [41,42]. Education (Edu) plays an important role in individual choice. People with less education receive less information about pandemics than people with higher education, which indicates that less educated groups are less likely to understand the importance of SI [43]. Personal behavior during an epidemic depends on an individual’s socioeconomic status (SS), as well as his or her perception of the epidemic in the community [44]. Married people enjoy better physical and mental health than people who are not married [45], so marital status may play a role in people’s behavior. Family members in need of special care (FNSC) are children, the elderly, those with disabilities, and those with chronic diseases. Respondents who live with people with these conditions have a higher risk perception concerning health [46,47,48]. Therefore, we assume that respondents with family members in need of special care have higher perceived risks. Furthermore, government trust (GT) [49,50,51], community resources (CRs) [52,53], and emergency services (ES) [49] are among the other variables affecting SI.

## 3. Materials and Methods

### 3.1. Study Area

SARS was first recognized in Guangdong Province, China, in November 2002, and the virus quickly spread; more than 7900 patients were infected in 30 countries [50]. This situation prompted us to explore the factors affecting the willingness of patients to self-isolate who may be infected in Guangdong Province. A market research company, LTD, which has a professional computerized database, was entrusted to carry out an online survey of pandemic prevention from 14 October to 3 November in 2018. The survey included the following steps. First, we determined the basic demographic characteristics such as gender, age, and education status of the respondents based on the 2017 Guangdong Internet Industry Development Report, which was published by the Guangdong Communications Administration. Second, a questionnaire was designed, which involved many aspects of SI. Third, 121 county-level units and 21 county-level city units were selected according to the statistical yearbook of Guangdong province 2018. Fourth, we transformed the designed questionnaire into a network questionnaire and sent it to the random sampling objects (2500 questionnaires) through a network link. Each interviewee was asked to provide a contact phone number. Finally, 2155 questionnaires were collected in Guangdong Province, which gives a gross response rate of 86.2% (2155/2500). After the survey, we made random checks of the respondents’ URLs and contacted some interviewees to ensure that every respondent was unique. Finally, we cleaned the data and obtained 1925 respondents; some subjects were excluded due to missing values. The net response rate was thus 77% (1925/2500). 

### 3.2. Definition and Measurement of Explanatory Variables

All of the variables were measured by the survey questions (see Table 1). 

We divided SN into two kinds of social pressure, from neighbors and from the health department, which were measured by the two questions below. An additional three questions were used to describe PBC, which include the individual and family’s risk perception of pandemics, and confidence to protect the family from a pandemic.

### 3.3. Data Analysis

We first examined the descriptive statistics of the variables and mapped the average scores of the willingness for SI using ArcGIS. Then, a correlation matrix was determined to examine these relationships and influences, in which 12 quantitative variables were included. Third, multiple linear regression models were adopted to explore the effects of ATT, SN, and PBC on SI. Finally, we explored the effects of these influencing variables on SI in different regions to test the stability of the results and identify the differences between regions. The statistical analysis was implemented using the statistical software Stata/MP, version 14.0 (StataCorp LP., Texas City, USA).

## 4. Results

Table 2 provides descriptive statistics for the dependent and independent variables. Overall, the average age of the participants was 35.12, with most between the ages of 18 and 66. Of the participants, 45.71% were female and 54.29% were male. Most of the participants had a college education or above, and 66.5% of them were married or cohabiting. In terms of economic status, most of the participants were in the middle class. The average FNSC was 2.025, which means at least two members per family were in need of special care. 

**Figure 2:**
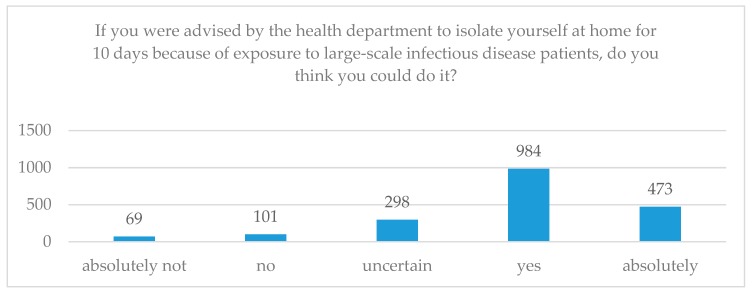
Willingness for SI.

The participants were asked: “If you were advised by the health department to isolate yourself at home for 10 days because of exposure to large-scale infectious disease patients, do you think you could do it?” Of those sampled, 8.83% said they could not isolate themselves, 75.69% said that they could, and 15.48% were unsure (see Figure 2).

### 4.1. Mapping SI

**Figure 3:**
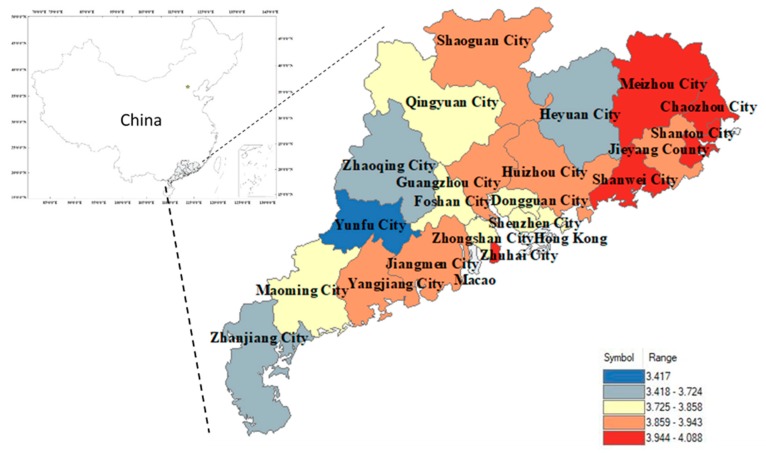
The mean score of SI reported in Guangdong, 2018.

The mean score of SI by municipality is mapped in Figure 3. Overall, the mean score of SI in Guangdong prefecture-level cities was between 3.4 and 4.1 out of 5, which indicates people have more willingness to self-isolate than they do in other regions. The cities exhibited some differences, with five cities (Shantou, Meizhou, Shanwei, Chaozhou, and Zhuhai) having a mean score greater than 4. Shantou City ranks first, with a mean score of 4.088, and Yunfu City ranks last, with a mean score of 3.417.

### 4.2. Influencing Factors of SI

Before a formal linear regression, the correlation matrix must be determined and the correlation of variables must be tested. These results are shown in Table 3, and a significant association was observed for 13 of the 16 indicators of SI. In particular, ATT, SN, and PBC were positively related to SI.

We first regressed on SI with the control variables as a benchmark model (1). Then, we added PBC, ATT, and SN sequentially to the model. Table 4 shows the crude and adjusted models for the multiple regressions of changes in the willingness to self-isolate when facing a large-scale infectious disease according to changes in PBC, ATT, and SN. The adjusted R^2^ of the benchmark model is lower than that of models (2)–(4), which means that those models are more appropriate than model (1) and that PBC, ATT, and SN are important factors of SI. Overall, respondents with a higher education level had a higher willingness to self-isolate when facing a pandemic.

PBC, ATT, and SN are the key factors influencing SI (significant at *p* < 0.001), and they have different effects according to the estimated coefficients. ATT has the most important effect on SI; when ATT increases by 1%, the willingness to self-isolate will increase by 0.443%. When SN increases by 1%, the willingness to self-isolate will increase by 0.162%. Finally, when PBC increases by 1%, the willingness to self-isolate will increase by only 0.082%.

### 4.3. Influencing Factors of SI in Difference Regions

The social-economic status in Guangdong Province is unbalanced, which manifests as differences in the population, economy, industrialization, and lifestyle. Guangdong Province is often divided into four parts: The Pearl River Delta and Eastern, Western, and Northern Guangdong (the Pearl River Delta includes Guangzhou, Shenzhen, Zhuhai, Foshan, Jiangmen, Dongguan, Zhongshan, Huizhou, and Zhaoqing; Eastern Guangdong includes Chaozhou, Jieyang, Shantou, and Shanwei; Western Guangdong includes Zhanjiang, Maoming, and Yangjiang; and Northern Guangdong includes Shaoguan, Heyuan, Meizhou, Qingyuan, and Yunfu) [51]. Regional differences may influence the willingness to self-isolate when facing a pandemic. Some researchers have explored the different influences of cancer risk [52], enteroviruses [53], epidemics [54], and diabetes mellitus [55], among other health issue, in these four parts. Therefore, we regressed on SI across different regions.

The coefficients and the significance levels of PBC, ATT, and SN in every model in Table 5 are similar to those in Table 4 (except the effect of SN is not significant in Western Guangdong). However, there are some regional differences. The control variables (including community resources, emergency services, and families with old people) have significant influences on SI in the Pearl River Delta. In Eastern Guangdong, age, education, socioeconomic, trust in leadership, and families with children have important influences on SI. However, only trust in leadership and families with old people have significant effects on SI in Western Guangdong. Moreover, SI in Northern Guangdong is only affected by age. The positive and negative influences of these control variables are similar to those in Table 4. No substantial changes in the independent and control variables can be detected, proving the robustness of our results mentioned above.

## 5. Discussions

In this paper, we built a conceptual model based on the TPB theory and regression method with Chinese provincial (Guangdong Province) data to investigate how ATT, SN, and PBC influence SI when facing a pandemic emergency.

### 5.1. The Factors Contributing to the Willingness to Self-Isolate

Theoretically, ATT, SN, and PBC are very different concepts; however, each plays an important role in social and behavioral research [26]. We ran multiple linear regression models to test the efficiency of the conceptual model we built based on the TPB. The coefficients of SI on ATT, SN, and PBC passed the significance test, and all were positive; these results were confirmed by the robustness checks, indicating that the TPB can be used to explain SI. Of the three considered factors, ATT plays the most important role, followed by SN and then PBC. The influence of SN on forming intention proved to be generally weaker in previous studies than the influence of ATT [56].

The coefficients of education on SI in models (1)−(4) were significant and positive, even if the significance levels are different. Therefore, when the participant’s educational level was higher, so was his or her willingness to self-isolate. In models (1) and (2), marriage had a significant and positive effect on SI, which means married individuals have a higher willingness to self-isolate. Socioeconomic status also had a significant and positive effect on SI in models (1) and (2), which means people of high socioeconomic status may have a higher willingness to self-isolate. As for government trust, trust leadership and trust government had significant positive impacts on SI, indicating that a higher degree of government trust promotes people’s willingness to self-isolate. Emergency services also had a significant positive influence on SI. Conversely, community resources are not useful for SI. One possible explanation is that if a community has more resources—such as money, food, and technology—the community will be more resilient, and people will be less willing to self-isolate because people believe that the community will address the risk of the infectious disease.

For family members in need of special care (children, old people, those with disabilities, or those with chronic diseases), the effects on SI of having a family member who is old or has a disability were significant and negative, which means such individuals may have less willingness to self-isolate. These people likely need to provide for the people in their family who need special care, and they have little chance to self-isolate. In contrast, the effect of having a family member with a chronic disease was significant and positive, indicating a higher willingness to self-isolate. Reasons for this finding may include the following: Vaccinations are recommended for those with chronic diseases, or those with a family member who has a chronic disease may have more medical knowledge and better understand the dangers of a pandemic. Those in a family with a child were less willing to self-isolate, but the effect was not significant.

### 5.2. Policy Recommendations for the Government

There may be a conflict of interest concerning the use of isolation, as measures must be balanced against the potential to compromise individuals’ liberty and autonomy [57]. Intention, PBC, ATT, and SN each reveal a different aspect of behavior and each can serve as a basis for attempts to change behavior [36]. Public health policies that encourage infected people to self-isolate can be beneficial to the community by lowering disease prevalence [58]. Behavioral science theory and research provide a perspective for understanding the factors contributing to people’s behavior. Consequently, the more we know about any given behavior, the more we can do to influence and change the behavior. Interventions to strengthen the willingness to follow SI instructions have timely relevance for the prevention and control of pandemic risk.

Based on the factors of SI, we found that, through theoretical and empirical analyses, our study identifies at least three important aspects that local governments need to consider when encouraging citizens to self-isolate when facing a pandemic. Our study has several implications for public health policy as well.

First, extensive publicity in various forms is necessary to help residents better understand the pandemic and raise awareness about early treatment when facing a pandemic risk. Greater understanding of pandemic influenza significantly increases compliance with public health containment measures [22]. However, some studies have indicated that SI does not solve all problems, and encouraging infected people to self-isolate does not always reduce the number of infections [58]. Therefore, we also need to classify the types of pandemic diseases and report to residents the correct solution to prevent a pandemic risk. It is of great importance to establish an early warning system to provide information about pandemics and to maintain communication with residents.

Second, the social norms of public health must be improved through joint efforts of the government, civil society, and the media. In contemporary political and legal life, law and policy play unique roles as two social norms and two means of social adjustment. On the one hand, relevant laws and regulations must be formulated to regulate behavior when facing a pandemic risk; on the other hand, public supervision and participation should be encouraged.

Third, although an influenza pandemic is perceived as a real risk in all countries, the level of self-efficacy appears to be rather low [59]. Therefore, when developing preparedness plans for an influenza pandemic, specific attention should be paid to risk communication and increasing perceived self-efficacy; otherwise, adherence to preventive measures may be low [59]. Therefore, residents should be given sufficient training to reduce the difficulty of applying health behavior through better public service.

In addition to the application of TPB, we found that some participants’ characteristics might also be factors related to SI. From the perspective of social assistance, residents have different demands; however, the characteristics of different people must be classified to make future social assistance more precise.

## 6. Conclusions

Voluntary home isolation and quarantine are effective and acceptable measures [8]; however, various factors may affect individuals’ willingness to self-isolate when facing a pandemic risk. In this article, we tried to show that the TPB provides a useful conceptual framework for SI when facing a pandemic risk. Using Chinese provincial (Guangdong Province) data, we investigated how ATT, SN, and PBC influence the willingness of self-isolate when facing a pandemic emergency. The results confirmed by the robustness checks show that ATT, SN, and PBC have significant positive influences on SI when facing a pandemic emergency, with ATT playing the most important role. Furthermore, we found that family members in need of special care have different effects on the willingness to self-isolate. The effects on SI of having a family member who is old or who has a disability are significant and negative, while the effect of having a family member with a chronic disease is significant and positive. These intentions, in combination with PBC, can account for a considerable proportion of variance in behavior. Based on these findings, we provided several implications for public health policy.

## Figures and Tables

**Table 1:** Variable description.

Variable	Indicators	Variable Description
SI	“If you were advised by the health department to isolate yourself at home for 10 days because of exposure to large-scale infectious disease patients, do you think you could do it?”	(1) Absolutely not, (2) No, (3) Uncertain, (4) Yes, (5) Absolutely
ATT	“Do you agree or disagree with the government’s mandatory isolation of infected people during a large-scale epidemic of an infectious disease?”	(1) Strongly disagree, (2) Disagree, (3) It does not matter, (4) Agree, (5) Strongly agree
SN	“If you were infected with a pandemic disease, would you let your neighbors or colleagues know?”	(1) Absolutely not, (2) No, (3) Probably, (4) Absolutely
	“How much do you trust the infectious disease prevention information/text messages issued by the health department?”	(1) Strongly distrust, (2) Distrust, (3) Cannot say trust or distrust, (4) Trust, (5) Strongly trust
PBC	“Do you think that a large-scale infectious disease would have a serious impact on you or your family?”	(1) Not serious at all, (2) Not too serious, (3) A little serious, (4) Very serious
	“Are you worried about large-scale infectious diseases?”	(1) Not worried at all, (2) Not too worried, (3) A little worried, (4) Very worried
	“Do you have confidence that you can protect yourself and your family if a large-scale infectious disease occurs?”	(1) No confidence, (2) Little confidence, (3) Uncertain, (4) Some confidence, (5) Very confident
Gender	“What is your gender?”	(1) Male, (2) Female
Age	“How old are you?”	(1) 18–44, (2) 45–59, (3) 60–74, (4) 75-89, (5) Older than 90
Education	“What is your highest education level (including your current level of study)?”	(1) Primary school and below, (2) Junior high school, (3) High school, (4) College, (5) Undergraduate, (6) Graduate and above
Marriage	“Are you married?”	(1) Not yet, (2) Cohabiting/married, (3) Separated/divorced, (4) Widowed
SS	“What level of social and economic status do you think you have?”	(1) Upper level, (2) Middle and upper levels, (3) Middle level, (4) Middle and lower levels, (5) Lower level
GT	“Do you agree or disagree with local leaders?”	(1) Strongly disagree, (2) Disagree, (3) It does not matter, (4) Agree, (5) Strongly agree
	“Do you agree or disagree that local government works well?”	(1) Strongly disagree, (2) Disagree, (3) It does not matter, (4) Agree, (5) Strongly agree
CR	“Do you agree or disagree that your community has the resources (capital/technology/materials/services, etc.) to solve community problems?”	(1) Strongly disagree, (2) Disagree, (3) It does not matter, (4) Agree, (5) Strongly agree
ES	“If an emergency happens, do you agree or disagree that your community can provide emergency services?”	(1) Strongly disagree, (2) Disagree, (3) It does not matter, (4) Agree, (5) Strongly agree
FNSC	“Is there anyone under 18 in your family?”	(1) Yes, (2) No
	“Is there anyone older than 60 in your family?”	(1) Yes, (2) No
	“Is there anyone with a disability in your family?”	(1) Yes, (2) No
	“Is there anyone with a chronic disease in your family?”	(1) Yes, (2) No

**Table 2:** Descriptive statistics of the variables.

Variable	Obs	Mean	Std. Dev.	Min	Max
SI	1925	3.878	0.958	1	5
PBC	1925	9.051	1.836	3	13
ATT	1925	4.004	0.915	1	5
SN	1925	7.387	1.194	2	9
Gender	1925	0.543	0.498	0	1
Age	1925	35.118	13.670	18	66
Edu	1925	4.242	1.202	1	6
Marriage	1925	0.665	0.472	0	1
SS	1925	3.079	0.941	1	5
TL	1925	3.576	1.032	1	5
TG	1925	3.787	0.979	1	5
CR	1925	3.594	0.963	1	5
ES	1925	3.729	0.947	1	5
Child	1925	0.740	0.439	0	1
Old People	1925	0.777	0.417	0	1
Disable	1925	0.124	0.330	0	1
Chronic	1925	0.384	0.487	0	1
FNSC	1925	2.025	1.002	0	4

TL: trust in leadership; TG: trust government.

**Table 3:** Correlation matrix of the studied variables.

Variables	SI	PBC	ATT	SN	Gender	Age	Edu	Marriage	SS	TL	TG	CR	ES	Child	Old Peo	Disable	Chronic
**SI**	1																
**PBC**	0.395 ***	1															
**ATT**	0.609 ***	0.371 ***	1														
**SN**	0.487 ***	0.332 ***	0.479 ***	1													
**Gender**	0.001	−0.028	−0.029	0.027	1												
**Age**	0.052 **	0.026	0.057 **	−0.011	0.411 ***	1											
**Edu**	0.069 ***	−0.009	0.032	0.080 ***	−0.066 ***	−0.316 ***	1										
**Marriage**	0.113 ***	0.083 ***	0.112 ***	0.077 ***	0.228 ***	0.620 ***	0.007	1									
**Soci Eco**	0.072 ***	−0.001	0.127 ***	0.017	0.011	0.118 ***	−0.349 ***	−0.049 **	1								
**TL**	0.203 ***	0.181 ***	0.151 ***	0.259 ***	0.036	−0.030	0.209 ***	0.115 ***	−0.271 ***	1							
**TG**	0.211 ***	0.148 ***	0.182 ***	0.308 ***	0.082 ***	0.003	0.175 ***	0.093 ***	−0.246 ***	0.624 ***	1						
**CR**	0.114 ***	0.131 ***	0.152 ***	0.199 ***	0.022	0.0150	0.129 ***	0.134 ***	−0.229 ***	0.472 ***	0.416 ***	1					
**ES**	0.234 ***	0.225 ***	0.242 ***	0.280 ***	0.014	0.001	0.080 ***	0.106 ***	−0.157 ***	0.453 ***	0.479 ***	0.537 ***	1				
**Child**	−0.012	0.083 ***	−0.009	0.020	−0.039 *	−0.035	0.065 ***	0.209 ***	−0.229 ***	0.139 ***	0.177 ***	0.093 ***	0.127 ***	1			
**Old peo**	−0.054 **	0.073 ***	−0.037	−0.020	0.024	0.075 ***	−0.105 ***	0.029	−0.066 ***	0.012	−0.007	0.017	0	0.100 ***	1		
**Disable**	−0.179 ***	−0.077 ***	−0.210 ***	−0.131 ***	−0.009	−0.086 ***	0.007	−0.103 ***	−0.172 ***	−0.024	−0.019	−0.003	−0.084 ***	0.119 ***	0.107 ***	1	
**Chronic**	−0.005	0.006	−0.011	−0.002	0.005	−0.023	0.059 **	0.020	−0.097 ***	−0.090 ***	−0.040 *	0.002	0.004	0.052 **	0.203 ***	0.276 ***	1

Standard errors in parentheses; * *p* < 0.05, ** *p* < 0.01, *** *p* < 0.001.

**Table 4:** The multiple linear regression models (*N* = 1925).

Variables	(1)	(2)	(3)	(4)
Gender	−0.073	−0.042	0.009	−0.009
	(−1.60)	(−0.98)	(0.26)	(−0.26)
Age	0.001	0.002	0.001	0.002
	(0.63)	(0.78)	(0.44)	(1.08)
Edu	0.055 **	0.063 ***	0.038 *	0.036 *
	(2.75)	(3.38)	(2.35)	(2.24)
Marriage	0.153 *	0.116 *	0.059	0.043
	(2.51)	(2.03)	(1.19)	(0.88)
SS	0.143 ***	0.124 ***	0.037	0.029
	(5.76)	(5.30)	(1.82)	(1.43)
TL	0.104 ***	0.067 **	0.068 **	0.059 **
	(3.79)	(2.62)	(3.06)	(2.69)
TG	0.109 ***	0.106 ***	0.056 *	0.024
	(3.86)	(3.99)	(2.42)	(1.04)
CR	−0.054 *	−0.049 *	−0.067 **	−0.069 **
	(−2.03)	(−1.97)	(−3.10)	(−3.25)
ES	0.163 ***	0.103 ***	0.047 *	0.036
	(5.88)	(3.95)	(2.05)	(1.61)
Child	−0.077	−0.111 *	−0.078	−0.064
	(−1.51)	(−2.33)	(−1.87)	(−1.59)
Old Peo	−0.079	−0.134 **	−0.093 *	−0.090 *
	(−1.55)	(−2.80)	(−2.24)	(−2.21)
Disable	−0.401 ***	−0.336 ***	−0.128 *	−0.109 *
	(−6.00)	(−5.35)	(−2.33)	(−2.03)
Chronic	0.128 **	0.112 **	0.062	0.052
	(2.84)	(2.65)	(1.68)	(1.45)
PBC		0.179 ***	0.098 ***	0.082 ***
		(16.49)	(9.82)	(8.30)
ATT			0.519 ***	0.443 ***
			(25.06)	(20.50)
SN				0.162 ***
				(9.86)
_cons	2.013 ***	0.832 ***	0.296	−0.239
	(9.94)	(4.11)	(1.67)	(−1.32)
*N*	1925	1925	1925	1925
*Adjusted R2*	0.129	0.237	0.426	0.454

Standard errors in parentheses; * *p* < 0.05, ** *p* < 0.01, *** *p* < 0.001.

**Table 5:** Regression on SI in the Pearl River Delta and Eastern, Western, and Northern Guangdong.

Variables	Pearl River Delta	Eastern Guangdong	Western Guangdong	Northern Guangdong
Gender	0.023	0.048	−0.007	0.036
	(0.43)	(0.74)	(−0.07)	(0.38)
Age	−0.004	0.007 *	−0.005	0.015 **
	(−1.55)	(2.07)	(−0.69)	(2.87)
Edu	0.016	0.055 *	0.051	0.018
	(0.64)	(2.15)	(1.02)	(0.32)
Marriage	0.085	0.135	−0.063	0.046
	(1.20)	(1.56)	(−0.40)	(0.38)
SS	−0.039	0.196 ***	0.101	0.020
	(−1.33)	(5.17)	(1.82)	(0.37)
TL	0.029	0.128 **	0.214 ***	0.027
	(0.88)	(3.23)	(3.53)	(0.46)
TG	0.063	−0.028	−0.058	0.051
	(1.87)	(−0.71)	(−0.83)	(0.83)
CR	−0.111 ***	−0.031	−0.003	0.022
	(−3.65)	(−0.80)	(−0.05)	(0.38)
ES	0.078 *	−0.002	−0.022	−0.031
	(2.40)	(−0.07)	(−0.31)	(−0.55)
Child	−0.052	−0.175 *	−0.084	0.015
	(−0.90)	(−2.43)	(−0.64)	(0.14)
Old peo	−0.107 *	0.028	−0.401 **	0.029
	(−1.97)	(0.35)	(−2.97)	(0.24)
Disable	−0.152	−0.091	−0.074	−0.066
	(−1.91)	(−0.81)	(−0.50)	(−0.55)
Chronic	0.070	−0.084	0.111	0.027
	(1.33)	(−1.37)	(1.02)	(0.27)
PBC	0.072 ***	0.062 **	0.096 **	0.077 **
	(5.23)	(3.31)	(3.14)	(2.72)
ATT	0.422 ***	0.421 ***	0.595 ***	0.374 ***
	(14.73)	(8.42)	(8.31)	(6.50)
SN	0.162 ***	0.193 ***	0.062	0.260 ***
	(6.71)	(6.62)	(1.20)	(6.55)
_cons	0.320	−0.94 1**	−0.323	−1.252 **
	(1.17)	(−2.80)	(−0.61)	(−2.69)
*N*	1107	350	224	244
*Adjusted R^2^*	0.407	0.568	0.565	0.636

Standard errors in parentheses; * *p* < 0.05, ** *p* < 0.01, *** *p* < 0.001.
